# Emotional “Patient-Oriented” Support in Young Patients With I–II Stage Breast Cancer: Pilot Study

**DOI:** 10.3389/fpsyg.2018.02487

**Published:** 2018-12-05

**Authors:** D. Di Giacomo, J. Ranieri, E. Donatucci, E. Perilli, K. Cannita, D. Passafiume, C. Ficorella

**Affiliations:** ^1^Department of MeSVA, University of L’Aquila, L’Aquila, Italy; ^2^San Salvatore Hospital, L’Aquila, Italy; ^3^Department of Biotechnological and Applied Clinical Sciences (DISCAB), University of L’Aquila, L’Aquila, Italy

**Keywords:** breast cancer diagnosis, psychological distress, anger, emotional impact of BC diagnosis, psychological treatment

## Abstract

**Objective:** The recent increased survival rate after breast cancer (BC) diagnosis and treatment is mostly related to early screening in younger age. Evidence gained from newly detected assessed psychological needs as well as certain emotional regulatory patterns in younger survivors has been related in the literature to an extremely low rate of adherence to the psychological therapies offered. Tailored psychological support is necessary. The aim of the present study was to verify the preliminary efficacy of supportive psychological intervention with an innovative orientation: the Early BC Psychological Intervention (EBC-Psy).

**Methods:** A controlled study design was used to investigate the efficacy of EBC-Psy intervention. Preliminary data involved twenty-four patients in the age range of 35–50 years, diagnosed with cancer at the early stage (I–II), who were exposed to the EBC-Psy intervention. To address the effect of intervention, emotional variables were tested before the treatment (Time 1) and then again after 6 months of the treatment (Time 2); evaluated emotional dimensions were anxiety, anger, depression, and psychological distress.

**Results:** EBC-Psy intervention appears to be effective on both depression (*p* = 0.02) and psychological distress (*p* = 0.01), even in a short time, highlighting the strength of a reinforced positive psychological conceptual approach to deal with the “disease condition” in younger patients; on the contrary, the control group evidenced an increase in the same emotional variables in timing.

**Conclusion:** Our findings, even if limited by this small-scale protocol, seemed to confirm the role of positive psychotherapy after BC diagnosis and treatment through the impact of cognitive processes, coping strategies, and psychological resilience. Future theoretical framework could boost the intervention to design an innovative survivorship model.

## Introduction

Breast cancer (BC) diagnosis is a traumatic event in a woman’s life with a sudden, strong impact on her quality of life. Several studies have detected and debated the negative effects of the diagnosis: decreasing the quality of affective relations, life expectation, long-term planning, productivity, and sociality. Signs of psychological difficulty such as depression, anxiety, anger, poor mood, social retraction, isolation, and aggression have been well documented (Burgess et al., 2005; Linley, 2006; Costanzo et al., 2007; Bennet et al., 2012; Lester et al., 2015; Ng et al., 2015; Conley et al., 2016; Gibbons et al., 2016). The primary negative impact is on quality of life as a woman; the secondary effect is simultaneously on her family and her social and working environments. Most studies that detected significant emotional suffering were conducted on aged populations (55-year-olds and older) as the incidence of BC increases with age (World Cancer Research Fund International ^1^). In recent decades, the characteristics of the population involved in BC clinical settings have changed. Early screening increased the chances of early detection of cancer diagnosis, improving early surgical and/or pharmacological interventions (less invasive, more conservative, and more positive survivorship outcomes), and involving patients at the ages younger than 55 (> 35-year-olds). These new protocols significantly increased the survival rate in patients with BC. The increased awareness in the research and clinical communities about the cancer experience can have positive outcomes, but it has also created new challenges: physical and mental impacts, and socioeconomic and cultural implications for patients (International Agency for Research on Cancer ^2^).

Moreover, clinical practice has demonstrated that changing the psychological needs of women increases their life expectations by longer survivorship: psychological resilience is the key to dealing with and overcoming oncological disease and self-perception as a patient with a chronic illness (Min et al., 2013; Molina et al., 2014; Quattropani et al., 2017). Di Giacomo et al. (2016) investigated the impact of psychological resilience in younger patients, analyzing their emotional distress. Findings showed that younger women felt powerless but not depressed and were well motivated to deal with their own clinical treatment protocols with good compliance, and they obtained fast, positive outcomes. Lately, some authors have extended such findings to the posttraumatic growth model of Tedeschi and Calhoun (2004): the posttraumatic growth is a construct whereby positive behavioral changes can be identified in dealing with and negotiating traumatic experiences (Giuliani et al., 2014). Moreover, Kolokotroni et al. (2014) analyzed the impact of psychosocial factors on posttraumatic growth after BC diagnosis. The authors highlighted the positive influence of the cognitive process and found that coping strategies are key factors for personal growth and for dealing positively with the cancer experience. Koutrouli et al. (2016) demonstrated the influence of social constraints on disclosure (deprivation of the opportunity to express feelings and thoughts regarding the trauma). In addition, the authors noted that the related cognitive processing of one’s own disease condition seems to play an important role in the development of posttraumatic growth, becoming more significant for younger patients toward reducing intrusions and reflective rumination. However, Parikh et al. (2015) maintained that patients with BC tend to experience posttraumatic growth but that psychological interventions should be implemented. In fact, only younger patients showed higher rates of dropout from psychological treatments. Brebach et al. (2016) conducted a meta-analysis investigating the uptake and adherence of cancer patients to psychological support. The adherence of patients with BC is quite low; half of all patients do not accept psychological support even though individual treatments of such services are offered. The factors influencing the uptake of intervention are distress measures, unmet needs, and the timing of the support. Research has found that patients with higher distress levels show lower rates of uptake and adherence, and an unmet need for psychological support despite declining it. Brebach et al. (2016) noticed that telephone interventions are more accepted than face-to-face interventions, and patients tend to agree more often to an intervention when it is offered by a nurse. Finally, the timing of access to the service is influential; the rate of adherence is higher when proposed close to the diagnosis.

Our study aimed to develop a tailored psychological intervention for younger patients with BC (age range 35–50) just after early medical treatment (surgical and/or pharmacological) using a cognitive approach tailored to their psychological needs after screened distress. We proceeded to plan a smart and innovative non-pharmacological treatment fitted to emotional weakness in young patients with BC: the Early BC Psychological Intervention (EBC-Psy). This psychological intervention has been oriented to a positive psychotherapy approach in order to develop an outcome of well-being in younger patients trying to win their life back. We conducted a controlled clinical study to verify the impact of the EBC-Psy in women in terms of efficacy after early and strong clinical treatments (scheduled medical protocols). The promising preliminary data are outlined below.

## Materials and Methods

### Ethics Statement

This study received Institutional Review Board (IRB) approval from University of L’Aquila, Italy (Prot. N° 15855/2015) and San Salvatore Hospital, L’Aquila, where participants were enrolled.

### Participants

Eligible participants were women who were 35 years old or older (< 50) living in Italy (the Abruzzo region, referring to L’Aquila’s healthcare). Participants had been enrolled in the medical oncology department of San Salvatore Hospital in L’Aquila, Italy. Inclusion criteria were as follows: (a) diagnosed with BC in stage I or II; (b) aged between 35 and 50; (c) just completed surgical treatment (mastectomy or lumpectomy); (d) underwent chemotherapy treatment; (e) not enrolled in another study; and (f) signed the informed consent statement.

The following exclusion criteria were used: (a) cancer recurrence; (b) metastasis; (c) preview mood/personality disorders; (d) signs of psychiatric disease; (e) psycho-pharmacological treatment; (f) age > 50; (g) abuse of alcohol or substance; and (h) being involved in ongoing psychological therapy in private/public services (Table 1).

**Table 1:** Demographic data of the sample.

	EBC-Psy group	Control group
**Education**		
Did not graduate	8.3%	8.3%
Graduated high school	66.7%	66.7%
Bachelor’s degree	25.0%	25.0%
**Relationship status**		
Married/living with partner	75.0%	75.0%
Single	8.3%	0.0%
Divorced/Separated	16.7%	25.0%
**Maternity**		
No children	16.7%	0.0%
One child	33.3%	25.0%
More than one child	50.0%	75.0%
**Occupation**		
Housewife	16.7%	16.7%
Employed	58.3%	58.3%
Self-employed	25.0%	25.0%
**Cancer stage**		
0	0.0%	0.0%
I	58.3%	50.0%
II	41.7%	50.0%
III	0.0%	0.0%
**Treatments^∗^**		
Mastectomy	50.0%	50.0%
Lumpectomy	50.0%	50.0%
Chemotherapy	8.3%^∗∗^	0.0%
Radiation therapy	0.0%	0.0%
Hormonal therapy	83.3%	100.0%
No treatment	8.3%	0.0%


*^∗^Treatments are not mutually exclusive. ^∗∗^Last session of chemotherapy at the moment of enrolment.*

### Study Design

Using these eligibility criteria, thirty-three patients with BC were approached for study enrolment during their last clinical visit. Of these, thirty (90%) agreed to participate and were enrolled. Patients who declined to participate cited external variables such as the facilities being too far away, the patient being unable to drive, and various family issues. Medical staff identified the eligible patients, who were then enrolled during check-up sessions, distributing them randomly into two groups; the medical staff was blinded as to which group had been exposed to the treatment. Participation in the study was voluntary. Information about patients was twofold: demographic data were compiled from self-reporting by patients, and we selected independent variables for inclusion in analysis if they were characterized according to age/life stage (e.g., having children, being employed, their marital status) and the variables were related to the cancer. Clinical data regarding the stage of BC (the TNM classification of malignant tumors), treatments, and therapies were collected from medical records.

A controlled clinical study was conducted by comparing treated and untreated groups to measure the psychological influence of the EBC-Psy.

**FIGURE 1:**
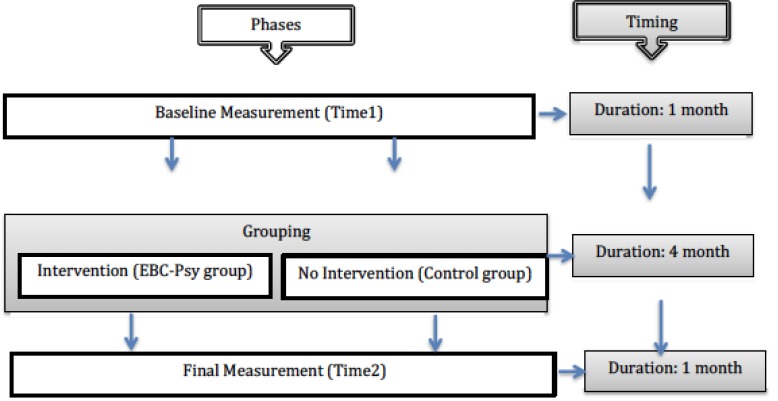
Study design representation.

Figure 1 shows the study design representation, distinguishing phases and timing.

**FIGURE 2:**
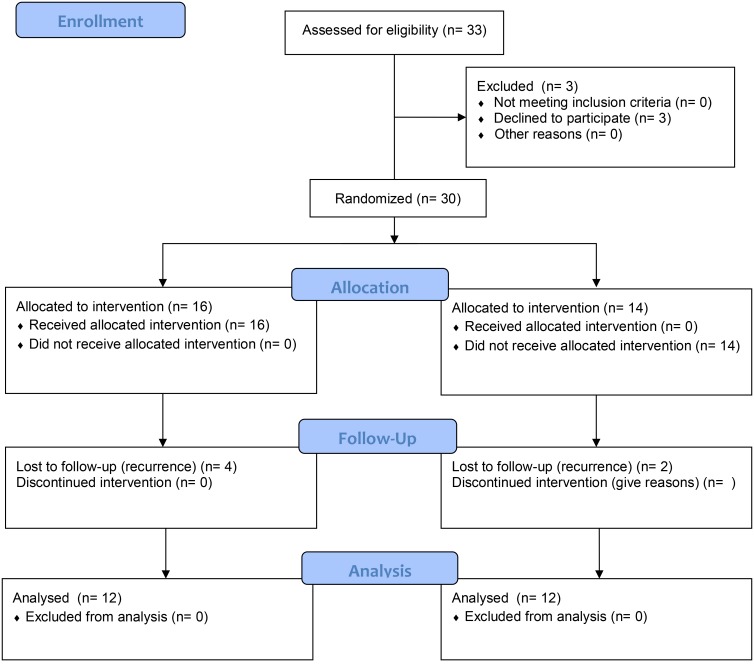
Representation of enrolment process by CONSORT Flow diagram.

Figure 2 represents the enrollment process.

The research design was divided into three phases: (1) baseline measurement, (2) grouping, and (3) final measurement. Eligible patients were approached to propose they take part in the experimental protocol (with or without psychological support), and, after acceptance, they were enrolled and distributed randomly in two groups: EBC-Psy group and Control group. The EBC-Psy group was composed of patients who took part in the whole experimental protocol (measurement and intervention phases), and the group was exposed to the EBC-Psy protocol for 4 months. The control group was composed of patients who only took part in the measurement phase of the research but not the intervention phase; it was considered the comparison group. Patients were measured by psychological testing at Time 1 and Time 2 over a 6-month period. All patients were enrolled after surgical and chemotherapy treatments (after 6–8 months from the diagnosis). The methods are consistent with CONSORT 2010 guidelines (Moher et al., 2001).

No payments were requested or given to take part in the experimental protocol.

### Training, Supervision, and Quality Control

The EBC-Psy was applied by a clinical psychologist with a cognitive-behavioral psychotherapeutic orientation who had been trained over a 2-week period; the training program was based on endpoints of the EBC-Psy intervention. The psychological measurement was administered by an experienced psychologist. Both psychologists were blinded as to the aim of the study. The scoring of the testing protocol was conducted by external blind judges. All EBC-Psy intervention sessions were recorded by digital camera; seven random sessions were used for internal quality control review and clinical supervision by a clinical team.

Medical staff members were involved in the enrolment of the sample and were in charge of managing the clinical path and check-up.

### Psychological Testing

The psychological measurement was conducted by self-reported testing to evaluate four emotional variables: anxiety, angry, psychological distress, and depression.

The following psychological tests were used: the State-Trait Anger Expression Inventory (STAXI) to measure the anger state, the State Trait Anxiety Inventory form Y (STAI-Y) to evaluate anxiety, the Psychological Distress Inventory (PDI) to assess distress, and the Beck Depression Inventory version 2 (BDI-II) to detect signs of depression.

#### State-Trait Anger Expression Inventory-2 (STAXI-2; Spielberger, 2004)

This is a self-administered questionnaire that aims to measure emotional states and personality traits; in particular, the evaluated traits are experience, expression, and control of anger. STAXI-II items are categorized into six scales—five subscales and an expression index. The experience of anger is conceptualized as having two components: State Anger (S-Ang) and Trait Anger (T-Ang). S-Ang is considered to be situational and refers to the level of anger that the respondent experiences during the assessment. T-Ang is defined as predisposition toward experiencing anger. The expression and control of anger are conceptualized as having four components: Anger Expression-Out (AX-O), Anger Expression-In (AX-I), Anger Control-Out (AC-O), and the Anger Expression Index (AX Index); the index provides an overall estimation of the anger expression and control scales. The scoring aims to reveal different personality traits related to anger risk. The internal reliability was α = 0.83 for the patient group and α = 0.61 for the control group.

#### State-Trait Anxiety Inventory-Form Y (Spielberger and Sydeman, 1994)

This is a self-reported test to measure state and trait anxiety. It is composed of forty items. The scoring is based on standard procedure. The internal reliability was α = 0.62 for the patient group and α = 0.73 for the control group.

#### Psychological Distress Inventory (Morasso et al., 1996)

This is a five-point self-administrated questionnaire that measures the impact of the disease and therapies in terms of psychological distress. It is composed of thirteen questions. The standard score indicates the presence/absence of psychological distress to measure global distress. This test was performed only in the patient group. The internal reliability was good (α = 0.86).

#### Beck Depression Inventory Version II (Beck et al., 1996)

This is a self-administered test. The BDI–II consists of twenty-one items to assess the intensity of depression in clinical and normal patients. Each item is a list of four statements arranged in order of increasing severity about a particular symptom of depression. The scoring indicates the presence/absence of depression and its relative degrees (from minimal to severe signs of depression). The internal reliability was good for both patient (α = 0.81) and control (α = 0.76) groups.

### Cancer Staging System

The method applied to classify the stage of cancer of the patients was the TNM, a cancer staging system developed by the American Joint Committee on Cancer (2010) and the Union for International Cancer Control. The TNM system is based on four main factors: (a) location of the primary tumor, (b) tumor size and extent, (c) lymph node involvement, and (d) presence or absence of distant metastasis. Basic data involved in staging the cancer are physical examinations, imaging tests, laboratory tests, pathology reports, and surgical reports. TNM staging is composed of the following stages: X (primary tumor cannot be evaluated), 0 (no evidence of primary tumor), IS (carcinoma in situ, that is, early cancer that has not spread to nearby tissue), and 1–4 (size and/or extent of the primary tumor). This cancer staging system was applied by clinical staff, and data were detected from recording files.

### Early Breast Cancer Psychological Intervention (EBC-Psy)

The EBC-Psy is a brief psychological intervention applied after the diagnosis of BC for young patients (35–50 age range) following primary clinical intervention in the healthcare system. It is aimed at the wellbeing of the young woman dealing with BC diagnosis and related medical treatments. The aim of the EBC-Psy is to enhance their emotional balance, overcoming the self-perception of “patient condition” after primary care and positive clinical outcomes. The innovation of the EBC-Psy intervention is to point out the experience of responding to adversity as an opportunity to experience positive change. In particular, the EBC-Psy is focused on one’s own emotional experience of the cancer and is based on the cognitive exploration of thoughts and feelings experienced as a result of cancer diagnosis and treatment. The EBC-Psy works to model in the patient an adjustment to a new perspective on life by taking care of one’s own affective and emotional priorities; through this cognitive exploration and focusing on thoughts related to specific psychological indicators (outlined below), the patient actively models her vision of life, boosting her positive perspectives and feelings.

The EBC-Psy is a supportive intervention to be applied just at the end of surgical and pharmacological treatments to provide the patient with psychological strategies to deal with being back in daily life through her cognitive analysis of negative feelings as a result of the cancer diagnosis. The scheduled protocol is based on individual sessions (60 min in duration) per week for 4 months (sixteen sessions in total). The cognitive approach shapes the EBC-Psy and is based on five indicators: overcoming the mental condition; recovering one’s personal perspective and expectations; changing one’s life vision and renewing oneself; body adaptation; and positive assertiveness. The psychological strategies are distinguished for each indicator. The indicator “overcoming the mental condition” is based on (a) reinforcing the individual opportunities, (b) readdressing the negative distorted cognitions, and (c) compensating for a dysfunctional underlying belief—each of which is respectively addressed by sharing and elaborating on one’s own experience, highlighting success in one’s own cancer illness experience, remembering the stress experienced related to the cancer diagnosis to manage the stress that was felt, and being aware of the feeling and emotional distress experienced by personalized strategies to improve the quality of mood. The indicator “recovering one’s personal perspective and expectations” is related to rediscovering personal characteristics and life priorities by a cognitive restructuring of the reaction after diagnosis and the processing of modified priorities in life, each based on the activation of a psychosocial context, particularly with family trained by the treatment team. The indicator “changing one’s life vision and renewing oneself” is related to an enhanced positive self-perception and a high compliance with a healthy lifestyle. The indicator “body adaptation” consists of strengthening the acceptance of body image through a cognitive and positive view of the modified body (body satisfaction, self-image, sexual issues). Finally, the “positive assertiveness” indicator works to modulate the fear reduction, balancing the positive and negative effects of one’s own experience, and an acceptance of the traumatic experience of BC by adjusting to the new reality; both tend to improve awareness of management strategies and provide opportunities to carry on with daily life.

Each session features four phases. The first phase is anamnesis and detection data: detection and collection of the information about the patient, her life, her experience, and her health condition. The second phase is the pre-clinical arrangement: the choice of psychological strategies based on the relevant and urgent needs of the patient. The third phase is a clinical-cognitive elaboration: adaptive processing by the exploration of negative feelings in order to boost self-awareness. The fourth phase is the closure of the clinical session, addressing the weaknesses and strengths of one’s own disease experience.

### Statistical Analyses

Descriptive statistics for baseline characteristics and the final measurement at each time point were calculated. A repeated measures MANOVA was used to detect the statistical significance of overall differences across the psychological variables when comparing emotional conditions of groups. A Kruskal-Wallis ANOVA test analyzed the effect of independent variables (the severity of cancer and typology of surgical interventions). The data were analyzed using the SPSS program with a fixed value α = 0.05.

## Results

There were 33 patients eligible for the present controlled study; the uptake rate was 90% (*n* = 30), and 9% of the patients declined at baseline measurement even though they had signed the informed consent. At final measurement, the dropout rate was 20% (*n* = 6); the reason was the modification of disease conditions (evidence for the recurrence of cancer). The adherence to the psychological path was high (90%).

Table 2 reports the raw scores (means and standard deviations) of psychological testing in Time 1 and Time 2.

**Table 2:** Raw scores (mean and standard deviations) of EBC-Psy and Control group performances on psychological test–retest evaluation (Time 1 and Time 2).

Variables	EBC-Psy group				Control group			
	Time 1		Time 2		Time 1		Time 2	
	*X*	*SD*	*X*	*SD*	*X*	*SD*	*X*	*SD*
**STAXI**								
*State Anger*	16.8	± 6.8	15.4	± 6.9	16.2	± 6.1	13.0	± 2.7
*Trait Anger*	21.3	± 4.8	19.8	± 4.9	20.9	4.9	19.3	±5.9
*Ax/out*	20.1	± 3.7	20.4	± 5.2	20.5	4.7	19.5	±4.7
*Ax/Con*	21.8	± 2.9	21.2	± 4.7	20.5	5.1	18.3	±4.2
*Ax/Ex*	30.2	± 6.5	29.5	± 10.8	30.1	7.1	30.7	±9.1
**STAI**								
*Total score*	88.4	± 10.7	83.1	± 5.9	88.4	± 10.7	89.0	± 3.1
**BDI**								
*Total score*	17.0	± 9.9	12.5	± 6.4	11.9	± 7.2	14.3	± 7.8
**PDI**								
*Total score*	34.9	± 11.0	28.3	± 5.3	31.8	± 7.0	33.2	± 5.5


First, we conducted a one-way ANOVA (2 groups × 8 measures), comparing the emotions of the EBC-Psy and control groups at the beginning of the treatment by assessing anxiety (STAI test), anger (STAXI test, composed of five sub-measures), depression (BDI), and psychological distress (PDI) variables. The statistical analysis showed no significant difference between groups. Looking at data and Graphic 1, both groups tended to experience the same emotions.

**GRAPHIC 1:**
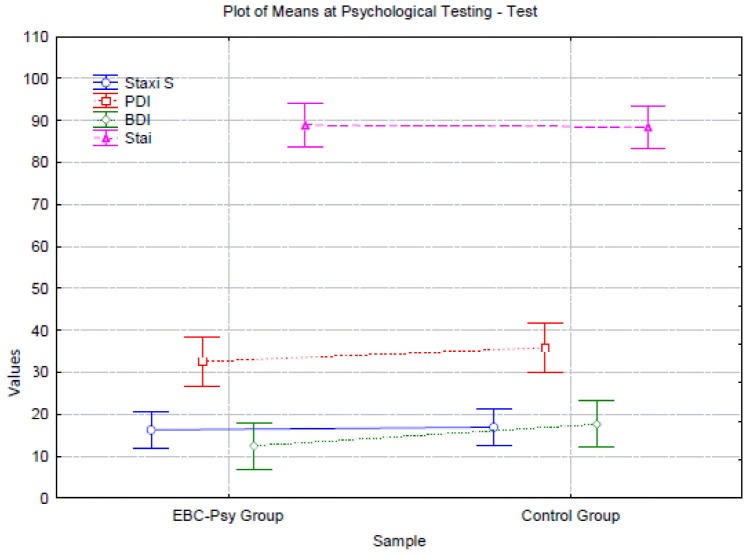
Representation of emotional condition of the participants at baseline measurement (Time 1).

We then conducted a MANOVA for repeated measures to verify the change over time after exposure to the experimental treatment in each group. We operated a MANOVA (2 × 2 × 2) for anxiety, depression, and psychological distress measures; then, a MANOVA (2 × 5 × 5) was conducted to analyze all variables of the anger measure. The statistical analysis evidenced significant differences in BDI (*d* = 0.8; *p* = 0.02) and PDI (*d* = 0.8; *p* = 0.018) measures.

In Graphics 2, 3, we report the representation of BDI and PDI performance.

**GRAPHIC 2:**
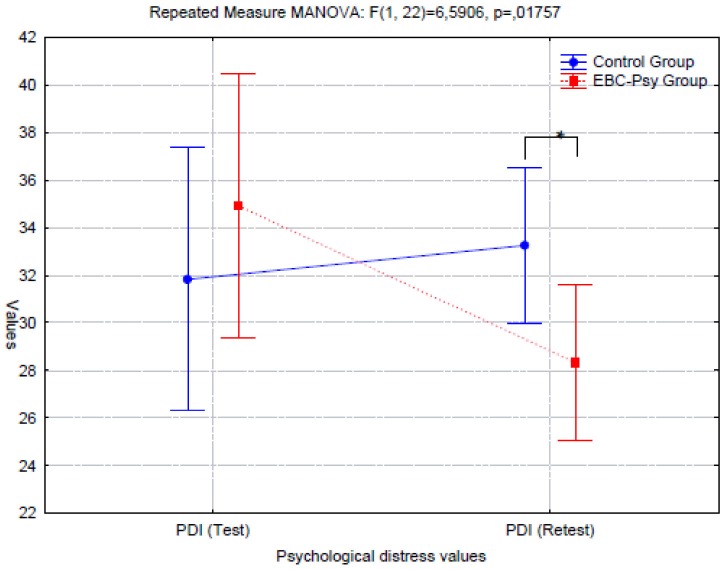
Representation of psychological distress conditions.

**GRAPHIC 3:**
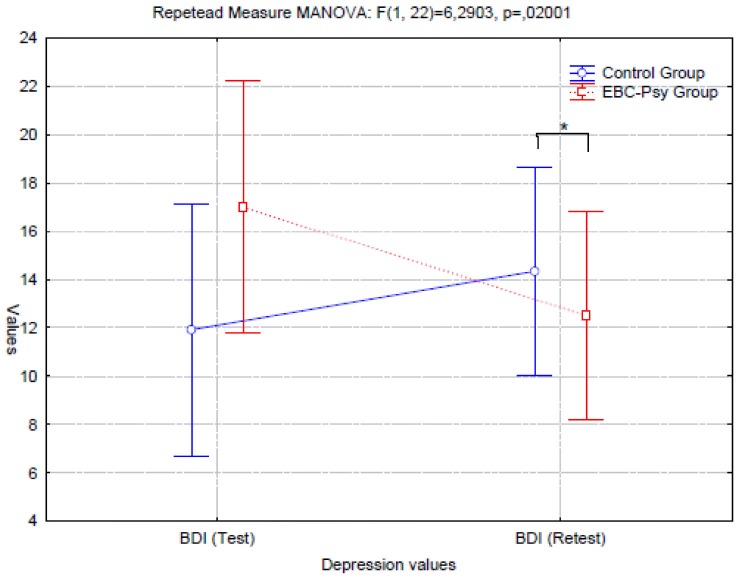
Representation of depression conditions (means and standard deviations).

Next, we conducted non-parametric analysis to verify the influence of cancer severity (TNM stage II and III) and surgical interventions (lumpectomy and mastectomy) on the decrease in psychological distress and depression at Time 2 in the EBC-Psy group. The Kruskal-Wallis ANOVA tests by variable TNM on PDI [H(1, *N* = 12) = 0.10 *p* = 0.74; X^2^ = 0.34 *p* = 0.55] and BDI [H(1, *N* = 12) = 2.41 *p* = 0.12; X^2^ = 3.08 *p* = 0.07] were not significant, nor was surgical intervention on PDI [H(1, *N* = 12) = 0.10 *p* = 0.70; X^2^ = 0.00 *p* = 1.00] and BDI [H(1, *N* = 12) = 0.78 *p* = 0.30; X^2^ = 1.33 *p* = 0.24].

## Discussion and Conclusion

The study investigated the impact of a new psychological intervention tailored for young patients with BC experience. A controlled clinical study design was used to propose an innovative psychological intervention to be applied after urgent surgical/pharmacological treatments. The EBC-Psy is a brief treatment based on emerging scientific findings about a new pattern of psychological needs from a population involved in intensive medical settings. Preliminary data demonstrated encouraging outcomes in this vulnerable population, highlighting the positive impact of the EBC-Psy. However, the study is still ongoing with a larger number of participants to finalize the results. At the same time, the participants who adhered to the clinical protocols have reduced their distress and depression in their daily activities. The keys of the EBC-Psy intervention on emerging psychological needs for new BC targets (younger patients) are the screening of psychological distress, well-timed intervention after diagnosis, and the shaping of coping strategies. The main goals of the intervention were to reduce distress and enhance treatment benefits. The positive outcome of the EBC-Psy intervention has been based on the reduction of signs of depression and psychological distress. Findings showed that the EBC-Psy favored patients overcoming self-perception as “long-term patients” just after the end of the clinical protocol of primary care. The women in the EBC-Psy group evidenced more positive perspectives to win back their lives thanks to a more efficient management of their own emotive balancing. On the contrary, the women in the control group showed increased distress and depression signs, and consequently experienced delayed and unbalanced wellness, likely related to a lower quality of life. In Di Giacomo’s studies (Di Giacomo et al., 2015, 2016; Di Giacomo, 2018), and confirmed in this research, two variables seemed to act as risk factors: (a) the reduction of the intensity and frequency of clinical sessions in the hospital (end of clinical treatments) and (b) positive responsiveness to the pharmacological protocols. Both factors fit with an increased level of distress in the patient due to the lower level of hospital stress and more negative thoughts about her own perspective on life (such as the possibility of recurrence and the risk of death). Against these risk factors, we focused on the experience of life, the changing events, and the awareness of psychological distress; they appear to be efficient counterpoints.

It will be interesting to evaluate the sustainability of the EBC-Psy intervention: actually, we are conducting follow-up evaluations 6 months from the end of the intervention and more around the 18th month after diagnosis.

Generally, our findings, even if limited by this small-scale protocol, seemed to confirm the positive psychotherapy role after BC diagnosis and treatment through the impact of the cognitive processes, coping strategies, and psychological resilience (Kolokotroni et al., 2014; Parikh et al., 2015; Di Giacomo et al., 2015, 2016; Koutrouli et al., 2016; Mattei et al., 2017a,b). In fact, they are considered key factors in personal change, in dealing positively with, and overcoming, the cancer experience. The cognitive process seems to play a preeminent role: in our EBC-Psy protocol, it worked efficiently to significantly reduce depression symptoms and psychological distress levels. Adjustment to life seemed to be the cognitive pathway to root the turning of positive behavior. The practical approach of the EBC-Psy could be in line with Tedeschi and Calhoun’s (2004) suggestion: “The frightening and confusing aftermath of trauma, where fundamental assumptions are severely challenged, can be fertile ground for unexpected outcomes that can be observed in survivors: posttraumatic growth” (p. 4). Our young women showed themselves to be powerful actors in their own lives, choosing to be anchored to real life and making their daily activity their strength. At this stage of the study, we had not yet tested the EBC-Psy as directly correlated to the posttraumatic growth model, but it could be considered an important and interesting future development of the framework.

The current study has some limitations that should be addressed in future research. First, the small sample size limits the generalization of the findings. Second, missing follow-up data limits our ability to examine the clinical relevance of the effects of intervention. Although our data are preliminary, we decided to discuss them because of their encouraging outcomes; the study is ongoing, and the sample size is increasing and will be larger over a longer period of time. In future works, we will address these points and analyze the relationship between intervention-induced positive growth and psychological response patterns by follow-up data. Moreover, it will be interesting to analyze the application of the posttraumatic growth model in the EBC-Psy framework.

## Future Developments

This study is a pilot study designed to address a specific psychological treatment for women to deal with the end of the clinical protocol of BC diagnosis and treatment. To evaluate the efficacy of the intervention, we must still conduct a randomized controlled trial in which we compare the EBC-Psy intervention to the counseling interventions that are currently applied in clinical protocols to verify the usability and specificity of the EBC-Psy. Moreover, our preliminary data are encouraging in terms of compliance and psychological strengthening, but we need other measures, controls, and data to detect its efficacy and then release the final version of the EBC-Psy; in other words, we are working to complete the validation process. However, sharing our research with the scientific community represents a valuable method to model and design better research planning, taking care to address the contributions (criticism and suggestions) of clinical professionals, and implementing the original study design.

## Author Contributions

DDG developed the study concept. All authors contributed to the study design. Testing and data collection were performed by JR. JR and ED performed the data analysis and interpretation under the supervision of EP. DP, KC, and DDG drafted the paper. All authors approved the final version of the paper for submission.

## Conflict of Interest Statement

The authors declare that the research was conducted in the absence of any commercial or financial relationships that could be construed as a potential conflict of interest.

## Footnotes

1 www.wcrf.org

2 www.iarc.fr
